# Supplementary material for: Housing Conditions Affect Adult Zebrafish (Danio rerio) Behavior but Not Their Physiological Status
Source: Animals (Basel). 2023 Mar 22;13(6):1120. doi: 10.3390/ani13061120 (PMC10044285; doi:10.3390/ani13061120)
Supplement: Supplementary file 1 [file animals-13-01120-s001.zip › TableS1.pdf]

Table S1. Significant data ($p < 0.05$) of the random factor batch from the novel tank test that are not represented in the text or figure. There were no interactions with the fixed factor treatment.

Variable of the NT	Factor	Description	Statistic test	*p*-value	Statistical test value[1]	Median [IQR] or Mean ± SD
Distance (m)	Batch	First vs. Second batch	Univariate analysis	0.004	Z= 27424.420	First:20.24 ± 3.61<br>Second: 13.78 ± 1.82
Average speed (m/s)	Batch	First vs. Second batch	Univariate analysis	0.010	Z= 4447.864	First:0.06 ± 0.01<br>Second: 0.04 ± 0.01
Distance (m) during erratic movements	Batch	First vs. Second batch	Univariate analysis	0.026	Z=603.722	First:14.75 ± 3.06<br>Second: 4.31 ± 1.98
Duration (s) of the erratic movements	Batch	First vs. Second batch	Univariate analysis	0.037	Z=296.714	First:170.83 ± 39.10<br>Second: 67.65 ± 24.57
Distance (m) swam in the BTM zone	Batch	First vs. Second batch	Univariate analysis	0.043	Z=221.917	First:17.43 ± 2.01<br>Second: 7.46 ± 1.06
Average speed (m/s) of enriched-housed fish in the UP zone	Batch	First vs. Second batch	Independent Samples T Test	0.022	t(3)= 4.418	First:0.08 ± 0.004<br>Second: 0.04 ± 0.007

[1] For Independent Samples t-test t(df)= t value. For Univariate analysis, presented is the standard score (Z); UP (upper) and BTM (bottom) zones of the tank; NT: Novel tank test.
