# Supplementary figures and images for: Housing Conditions Affect Adult Zebrafish (Danio rerio) Behavior but Not Their Physiological Status
Source: Animals (Basel). 2023 Mar 22;13(6):1120. doi: 10.3390/ani13061120 (PMC10044285; doi:10.3390/ani13061120)

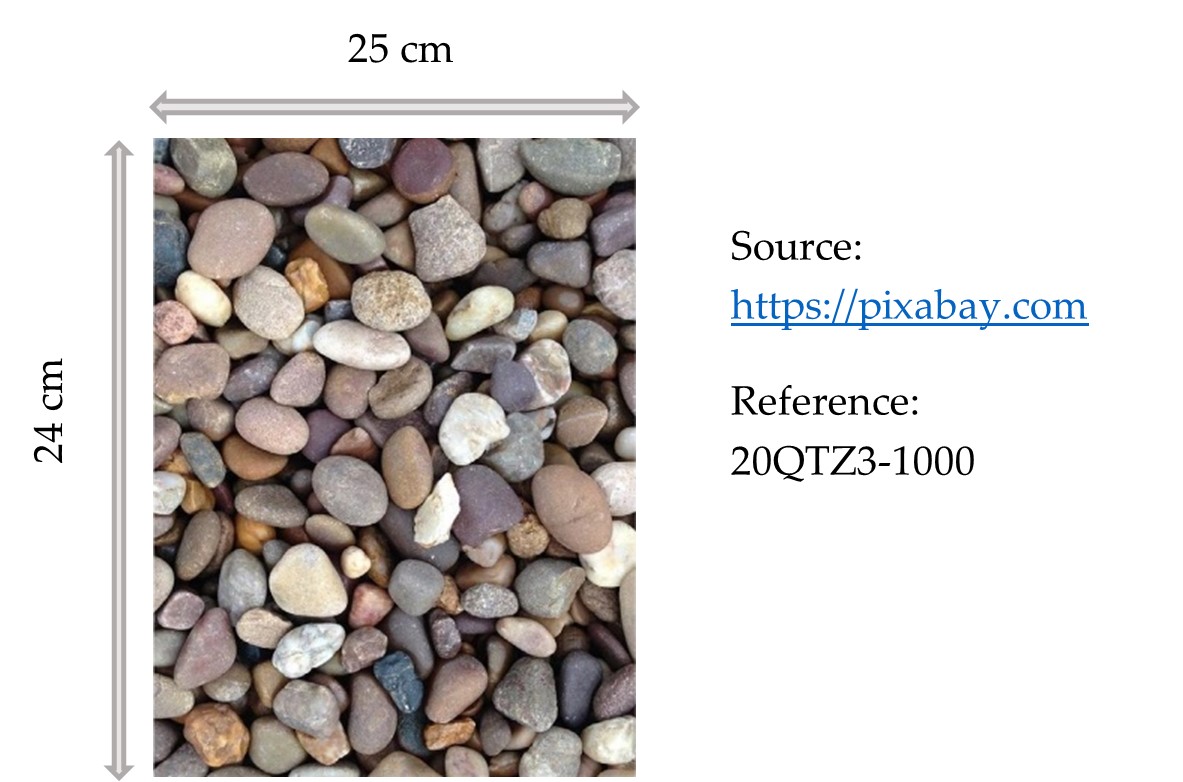

Supplement: Supplementary file 1 [file animals-13-01120-s001.zip › Figure S1.jpg]

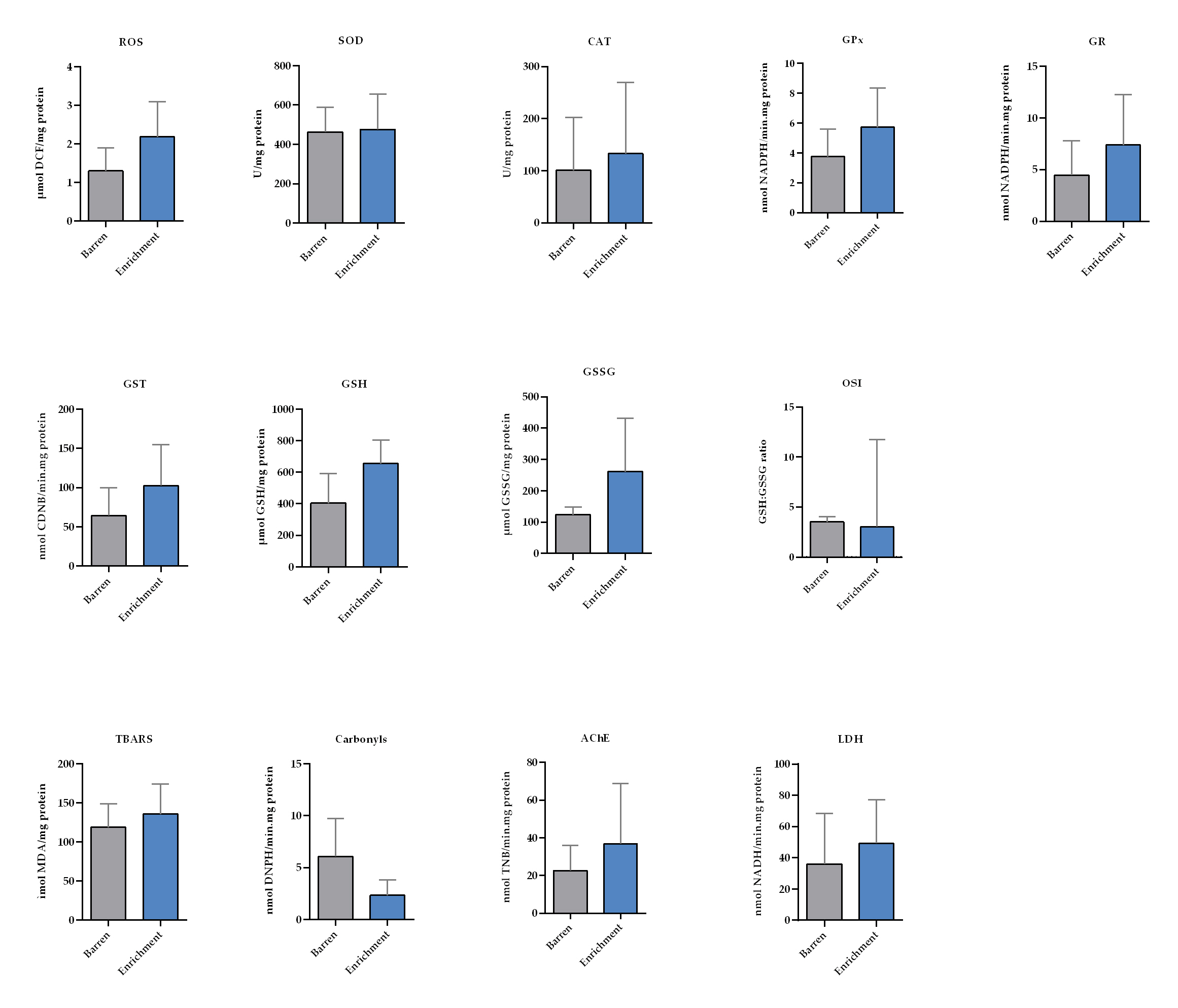

Supplement: Supplementary file 1 [file animals-13-01120-s001.zip › Figure S2.jpg]
